# Supplementary material for: A Simple and Effective Method to Concentrate Hepatitis C Virus: Aqueous Two-Phase System Allows Highly Efficient Enrichment of Enveloped Viruses
Source: Viruses. 2022 Sep 8;14(9):1987. doi: 10.3390/v14091987 (PMC9503063; doi:10.3390/v14091987)
Supplement: Supplementary file 1 [file viruses-14-01987-s001.zip › viruses-1906523-supplementary.pdf]

Supplementary Materials

For

A Simple and Effective Method to Concentrate Hepatitis C Virus: Aqueous Two-Phase System Allows Highly Efficient Enrichment of Enveloped Viruses

Hee Sun Kim [1‡], Johan Yi [2‡], Jinbae Yu [3], Jaesung Park [2, 4*] and Sung Key Jang [1,3*]

[1]POSTECH Biotech Center, Pohang University of Science and Technology, Pohang 37673, Gyeong-buk, Korea

[2]Department of Mechanical Engineering, Pohang University of Science and Technology, Pohang 37673, Gyeong-buk, Korea

[3]Department of Life Sciences, Pohang University of Science and Technology, Pohang 37673, Gyeong-buk, Korea

[4]School of Interdisciplinary Bioscience and Bioengineering, Pohang University of Science and Technology, Pohang 37673, Gyeong-buk, Korea

[*]Correspondence: sungkey@postech.ac.kr (S. Jang); jpark@postech.ac.kr (J. Park)

[‡]These authors contributed equally to this work.

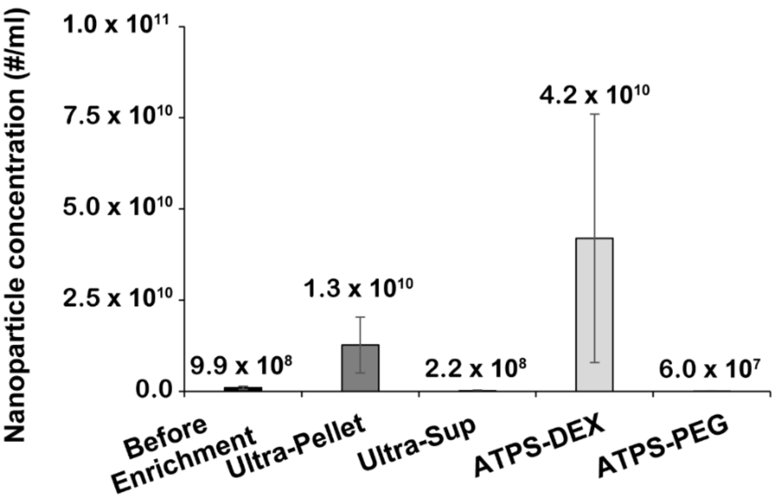

Supplementary Figure S1. Analysis of nanoparticles concentrated by ultracentrifugation and the aqueous two-phase system (ATPS) detected by nanoparticle tracking analysis (NTA). The nanoparticle concentration was measured using nanoparticle tracking analysis (NTA). HCV (JC1-E2-Flag variant) particles were concentrated by either ultracentrifugation or the ATPS. The columns and bars in panels represent the means and standard deviations, respectively. Experiments were performed three times with six times of particles tracking for each experiment. ATPS-DEX: ATPS dextran layer; ATPS-PEG: ATPS polyethylene glycol (PEG) layer.

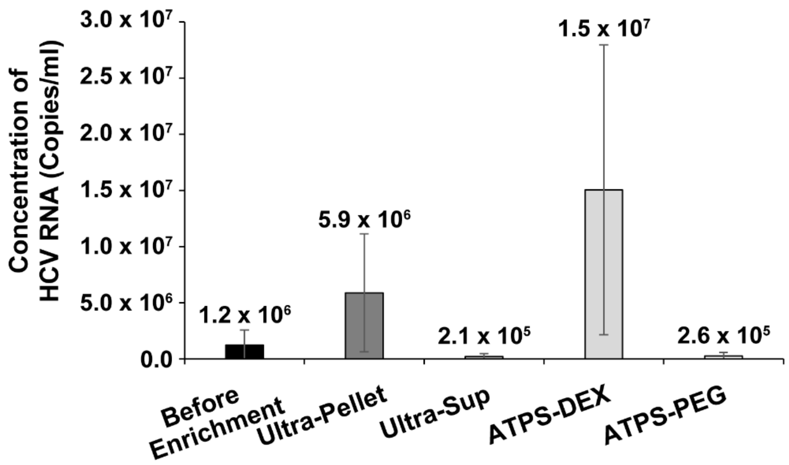

Supplementary Figure S2. The efficacies of HCV RNA enrichments by ultracentrifugation and ATPS. The concentration of HCV (JC1-E2-Flag variant) RNAs in samples before and after enrichment processes (ultracentrifugation and ATPS) were quantified using quantitative reverse transcription polymerase chain reaction. The relative concentrations of HCV RNAs in solutions before and after enrichment are depicted. The average value is depicted on the top of each column. Experiments were performed three times. ATPS-DEX: ATPS dextran layer; ATPS-PEG: ATPS polyethylene glycol (PEG) layer.

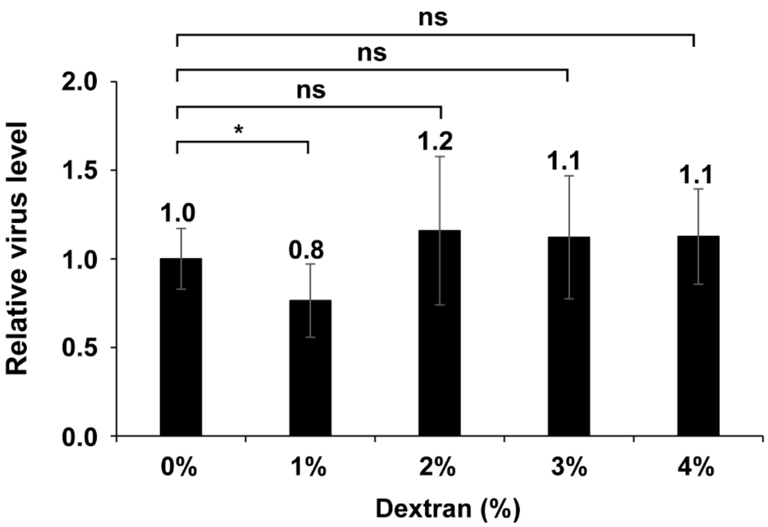

Supplementary Figure S3. Effect of dextran on hepatitis C virus (HCV) infectivity. The Huh-7.5.1 cells were infected with HCV (JFH-5a Rluc) in the presence of varying amounts of dextran (0 % - 4 %). After 3 days of infection, the levels of HCV proliferation were monitored by measuring Renilla luciferase activity in the cells. Experiments were performed three times in duplicate. The columns and bars represent the means and standard deviations, respectively.
